# Timing of Intervals Between Utterances in Typically Developing Infants and Infants Later Diagnosed with Autism Spectrum Disorder

**DOI:** 10.3390/brainsci15080819

**Published:** 2025-07-30

**Authors:** Zahra Poursoroush, Gordon Ramsay, Ching-Chi Yang, Eugene H. Buder, Edina R. Bene, Pumpki Lei Su, Hyunjoo Yoo, Helen L. Long, Cheryl Klaiman, Moira L. Pileggi, Natalie Brane, D. Kimbrough Oller

**Affiliations:** 1 School of Communication Sciences and Disorders, University of Memphis, Memphis, TN 38152, USA; zprsrush@memphis.edu (Z.P.); ehbuder@memphis.edu (E.H.B.); ebene@memphis.edu (E.R.B.); 2 Spoken Communication Laboratory, Marcus Autism Center, Children’s Healthcare of Atlanta, Atlanta, GA 30329, USA; gordon.ramsay@emory.edu (G.R.); cheryl.klaiman@emory.edu (C.K.); moira.pileggi@choa.org (M.L.P.); natalie.brane@choa.org (N.B.); 3 Department of Pediatrics, Emory School of Medicine, Atlanta, GA 30322, USA; 4 Department of Mathematical Sciences, University of Memphis, Memphis, TN 38152, USA; cyang3@memphis.edu (C.-C.Y.); 5 Institute for Intelligent Systems, University of Memphis, Memphis, TN 38152, USA; 6 Department of Speech, Language, and Hearing, School of Behavioral and Brain Sciences, The University of Texas at Dallas, Richardson, TX 75080, USA; lei.su@utdallas.edu (P.L.S.); 7 Callier Center for Communication Disorders, The University of Texas at Dallas, Dallas, TX 75235, USA; 8 Department of Communicative Disorders, College of Arts & Sciences, The University of Alabama, Tuscaloosa, AL 35487, USA; hyoo3@ua.edu (H.Y.); 9 Communication Sciences Program, Department of Psychological Sciences, Case Western Reserve University, Cleveland, OH 44106, USA; helen.long@case.edu (H.L.L.); 10 Konrad Lorenz Institute for Evolution and Cognition Research, A-3400 Klosterneuburg, Austria

**Keywords:** infant vocalizations, autism spectrum disorder (ASD), temporal organization, sex differences

## Abstract

**Background:** Understanding the origin and natural organization of early infant vocalizations is important for predicting communication and language abilities in later years. The very frequent production of speech-like vocalizations (hereafter “protophones”), occurring largely independently of interaction, is part of this developmental process. **Objectives:** This study aims to investigate the gap durations (time intervals) between protophones, comparing typically developing (TD) infants and infants later diagnosed with autism spectrum disorder (ASD) in a naturalistic setting where endogenous protophones occur frequently. Additionally, we explore potential age-related variations and sex differences in gap durations. **Methods:** We analyzed ~1500 five min recording segments from longitudinal all-day home recordings of 147 infants (103 TD infants and 44 autistic infants) during their first year of life. The data included over 90,000 infant protophones. Human coding was employed to ensure maximally accurate timing data. This method included the human judgment of gap durations specified based on time-domain and spectrographic displays. **Results and Conclusions:** Short gap durations occurred between protophones produced by infants, with a mode between 301 and 400 ms, roughly the length of an infant syllable, across all diagnoses, sex, and age groups. However, we found significant differences in the gap duration distributions between ASD and TD groups when infant-directed speech (IDS) was relatively frequent, as well as across age groups and sexes. The Generalized Linear Modeling (GLM) results confirmed these findings and revealed longer gap durations associated with higher IDS, female sex, older age, and TD diagnosis. Age-related differences and sex differences were highly significant for both diagnosis groups.

## 1. Introduction

Understanding the origin and natural organization of infant vocal development is important for predicting communication and language abilities in later years [1,2,3,4,5,6]. The tendency of infants to produce speech-like vocalizations, known as “protophones” [7], at very high rates appears to play a pivotal role in this development [8,9,10]. The production of protophones has been argued to be a requirement for language development since language requires the learning of new categories of sound not in the human innate repertoire (which consists, for example, of cries and laughs). Protophones include three main phonatory categories: vocants (vowel-like vocalizations), squeals (high-pitched vocalizations), and growls (low/mid-pitched vocalizations that usually show harsh or fry phonation) [7]. Infants produce protophones from the first week of life [7,11]. In human coding of all-day recordings, thousands of these protophones are observed daily in infants across the whole first year [12], both in typically developing (TD) infants and those later diagnosed with autism spectrum disorder (ASD) and other conditions.

Most protophones produced by infants in the first year of life are endogenous, i.e., not elicited by caregivers [13,14]. The pattern of production of infant sounds is not, however, random. The endogenously produced protophones tend to be grouped together in phrase-like sequences, or “clusters” [15,16]. Using data from an automated analysis of all-day recordings, a clustering pattern was found in interaction sessions for infant utterances with respect to each other in both infant speech-related (i.e., protophones) and infant non-speech-related (i.e., cries and other non-speech-like) utterances. In addition, ref. [16] demonstrated that as the time scale of analysis was lengthened, clustering patterns were easier to detect, suggesting a hierarchical organization of timing by the infant. The authors interpreted this pattern as evidence that vocalizations are not randomly distributed but are temporally organized.

### 1.1. Relevant Studies on the Timing of Utterance Intervals

We know of no research indicating the distribution of durations separating infant protophones that are clustered in all-day naturalistic recordings where endogenous protophones most representatively occur; however, some research studies briefly report the timing between vocalizations of TD infants during mother–infant interaction [2,17,18,19,20]. Ref. [19] measured the mean of infant “intrapersonal pause,” defined as the silence interval between the end of an infant’s spontaneous vocalization and the beginning of the next spontaneous vocalization produced by the same infant during a 9-month laboratory visit. The mean of intrapersonal pauses during 5 min naturalistic toy-play with the mother for low-risk infants (LR, who had no family history of ASD) and high-risk infants (HR, who had an older sibling diagnosed with ASD) was about 2 and 1.8 s, respectively.

Ref. [18] investigated the duration of within-turn pauses (presumably equivalent to intrapersonal pauses) in infants, where the same infant produced the first as well as the second utterance in free-play mother–infant interactions. The results showed a positive correlation between age and within-turn pause durations. Infants showed longer within-turn pauses after their own vocalizations at older ages, suggesting an indicator of advancing communication skills.

Ref. [20] found the mean duration of infants’ within-turn pauses to be 745 milliseconds (ms), with a standard deviation (SD) of 557 ms during mother–infant interaction. The authors considered a within-turn pause of less than 300 ms to be within an utterance rather than consisting of a pause. No pause of longer than 3000 ms was included in their data, so the effective range of within-turn pauses was 300 to 3000 ms. They did not find significant differences between within-turn pauses based on age or sex.

Ref. [2] found the average duration of pauses between vocalizations of infants (intrapersonal pauses) in various interaction settings, mother–infant, and stranger–infant home turn-taking. The results showed the mean duration of intrapersonal pauses for infants in home interaction sessions with mothers was 660 ms (SD = 320). In addition, the mean duration of intrapersonal pause for infants in interaction sessions with strangers was 520 ms (SD = 250). The authors considered a within-turn pause of less than 250 ms to be within an utterance rather than consisting of a pause. Notably, Jaffe et al. [2] used an entirely automated method to determine durations of intrapersonal pauses for vocalizations of infants and other speakers; the procedure may often have been mistaken in the identification of utterances. Their system appeared to treat cries and whimpers in the same way as speech-like vocalizations because the researchers did not exclude crying or fussing until it had lasted at least 30 s. Table 1 shows a summary of prior studies assessing the mean of intrapersonal pauses (equivalent to gaps in our terminology) in infancy in interaction recordings. The large differences between mean pause durations appear to be due to differences in the maximum time allowed within a single conversation or monologue.

It is evident that if the maximum duration of the time frame for measured intrapersonal pauses is decreased, the mean intrapersonal pause is necessarily shortened. Northrup and Iverson (2015) [19] likely had a long maximum duration or no maximum duration, whereas Gratier et al. (2015) [20] had a 3000 ms maximum. We do not have information about Jaffe et al. (2001) [2], but they presumably had a similar maximum to Gratier et al. (2015) [20], since the means were similar.

The studies in Table 1 measured the silence interval between the end of a TD infant’s vocalization and the beginning of the next vocalization produced by the same infant. In another study, Bateson (1975) [17] reported the mean (3140 ms, SD = 427) between the start of an infant’s vocalization and the start of the next vocalization produced by the same infant at the ages of 49 to 105 days across all observed home recordings of mother–infant interactions. This value presumably includes both the average duration of utterances and the average duration of gaps between them.

### 1.2. The Present Study and Research Questions

Previous research mostly focused on reporting the timing between infant vocalizations during mother–infant interactions in a laboratory setting, leaving a gap in our understanding of the clustering organization of infant protophones in more naturalistic settings, where protophones are usually not elicited, but are instead endogenous and independent of other speakers. Thus, we aim to explore how gap durations (offset-to-onset intervals) between infant protophones are distributed in naturalistic settings, and how gap durations may interact with diagnosis, age, sex, and infant-directed speech (IDS). The distribution of gap durations between infant protophones in naturalistic settings may offer important insight into how speech in its most primitive form is temporally organized. In addition, it may be important to determine the extent of variation among such distributions, especially in cases where language delay or disorder may occur (in ASD, for example). This exploration could hold the potential to deepen our comprehension of early communicative markers associated with ASD. For this proposed exploratory study, the research questions are:

1. What is the distribution of gap durations (offset-to-onset intervals) between protophones in TD infants, and are there differences in the distribution of gap durations between segments with higher and lower amounts of IDS?
2. Are there differences in the distribution of gap durations between TD and autistic infants across recording segments with higher and lower IDS?
3. Are there differences in the distribution of gap durations across age groups in both TD and autistic infants?
4. Are there differences in the distribution of gap durations across sexes in both TD and autistic infants?
5. Are there potential interactions between diagnosis (TD vs. ASD), IDS (higher vs. lower), sex, and age in gap durations?

## 2. Materials and Methods

### 2.1. Participants

Our study was based on a collaborative effort between the University of Memphis, Emory University in Atlanta, and the Marcus Autism Center in Atlanta. Longitudinal all-day audio recordings were collected using Language ENvironment Analysis (LENA, [21,22]) recording devices in the homes of infants recruited through the Marcus Autism Center. All the recordings were collected at the Marcus Autism Center, supervised by the second author. Our sample is a subset of 312 infants for whom human coding of the recordings was carried out at the University of Memphis under the supervision of the last author. 124 of the 312 infants were at high risk (HR) for an ASD diagnosis because they had an older sibling diagnosed with ASD, and the remainder were at low risk (LR) because they had no family history of ASD. A total of 147 of the 312 infants were included in our study: 103 TD infants (56 males and 47 females) and 44 autistic infants (29 males and 15 females). Infants were assessed at 2 years of age, and all HR and LR infants with clinical features were brought back for evaluation at 3 years old. The assignment to the TD and ASD groups was based on final assessments, which included the Autism Diagnostic Observation Schedule (ADOS-2; [23]) and independent clinical evaluations by two expert clinicians at the Marcus Autism Center. 65% of TD infants and 25% of autistic infants were designated as having high socioeconomic status (SES) based on maternal education level, known to be the best single predictor of SES relevant to speech and language [24].

### 2.2. Procedure

A total of 1259 recordings, each lasting ~11 h, were collected in the homes of infants, approximately once a month from birth, as part of an NIH-funded Autism Center of Excellence (NIH P50 MH100029) conducted at the Marcus Autism Center. For more details about recordings, see [12,15,25]. The Origin of Language Laboratory in Memphis (OLL) contributed human coding of the infant recordings as part of the collaborative longitudinal study (NIH DC015108). English-speaking graduate students in Speech-Language Pathology or Audiology were trained for 6 to 8 weeks to code the recordings. They typically entered training at the beginning of their master’s or AuD degree program and coded for two years. Over a seven-year period, there were usually 6–12 coders on the team, with a grand total of 36 who contributed to the database.

In Phase 1, the coding team counted the frequency of protophones (principally growls, squeals, and vocants) in 21 randomly selected 5 min segments of each daily recording in real time using Action Analysis, Coding, and Training software (AACT; [26]). AACT is a time-aligned coding tool that displays both waveforms and spectrograms in real time and allows audio playback with a cursor tracking the location in the recording. Coders used AACT to designate the appropriate category of utterances as they occurred using both auditory information and the acoustic displays. In Phase 2 coding, AACT selected 8 of the 21 segments based on high infant protophone rate as determined by the Phase 1 coding. In Phase 2, canonical and non-canonical syllables of the infant and vocalizations of speakers other than the infant wearing the LENA recorder were counted by coders in real-time. Finally, in Phase 3 coding, AACT automatically selected 2 of the 8 segments from Phase 2 for repeat listening coding based on two criteria: (1) high volubility of infant vocalization and relatively low IDS, and (2) high volubility of infant vocalization and relatively high IDS (See the Supporting Information of [25] for the algorithm specifying the Phase 2 and Phase 3 segment selection process). In Phase 3, the same coders identified onsets and offsets of each infant utterance along with a designation of the appropriate category of utterances (vocant, squeal, growl, cry, and whimper). As a result, two 5 min segments were human coded from each of the 1259 recordings. For more details about the coders, coding environment, and coding agreement, see [15,25]; see Figure 1 in [25] for a schematic diagram of the multi-phase coding procedure.

For the analysis of timing in the present study, we used approximately 1500 five min segments of Phase 3 data. In these segments, infants produced over 90,000 protophones according to the human coders, for which both the protophone durations and the gap durations were determined. Intervening utterances of other speakers as well as cries and whimpers were simply ignored (see Supplement S1). Agreement data for utterance durations were determined for the same dataset in a prior study [12], where the average absolute value difference between utterance durations of two independent coders was determined to be approximately 4%.

### 2.3. Data Analysis

In the analysis of the present study, we first evaluated gap durations using Generalized Sequential Querier software (GSEQ, Version 5.1; [27]) and R software (Version 4.5.0; [28]). Our criterion for the whole-time interval to be considered in the gap duration analysis was 75 to 10,000 ms. All short gap durations < 75 ms (only about 0.1% of gap durations in the 0–10,000 ms interval were <75 ms) were removed from the data at the point of analysis since such short gap durations should have been treated as within-utterance events in accord with the coding training criteria. We also reasoned that after a 10 s gap duration, a new cluster should begin. This 10 s criterion was not based on previous literature because no study has established a standard threshold to define clusters of infant vocalizations. Instead, it was chosen as a practical criterion for exploratory purposes that presumably includes multiple levels of temporal organization in infant vocalizations, as suggested in the hierarchical clustering framework proposed by [13].

Two analytical approaches were used: (1) To examine whether the overall distributions of gap durations differed significantly across groups, we conducted non-parametric Kolmogorov–Smirnov (K-S) tests. The K-S test determines significant differences between two probability distributions which were non-normal, and can differentiate between distributions of different shapes.

K-S tests do not, however, provide the opportunity to test various factors simultaneously against each other. (2) To assess the potential interaction effects between diagnosis (TD vs. ASD), IDS (higher vs. lower), sex, and age on gap durations, we used Generalized Linear Models (GLMs) with a Gamma distribution and a negative inverse link function. GLMs enabled us to model group-level effects and to investigate potential interactions among the different factors. The GLM method also provides a statistical estimate of effect size based on predicted means and standard deviations for distributions.

## 3. Results

### 3.1. Analysis of Distribution of Gap Durations Using Kolmogorov–Smirnov Tests

In our infant-level analysis, the histograms display the estimated probability density of gap durations on the y-axis. To ensure that individual infants did not disproportionately influence the overall distributions, we assigned a weight to each data point based on the proportion of gap durations contributed by each infant. This weighting approach balanced the contribution of each infant.

#### 3.1.1. Gap Durations (Offset to Onset Intervals) Between Protophones in TD Infants and Breakdown of Results Across Higher and Lower IDS Recording Segments

**Figure 1:**
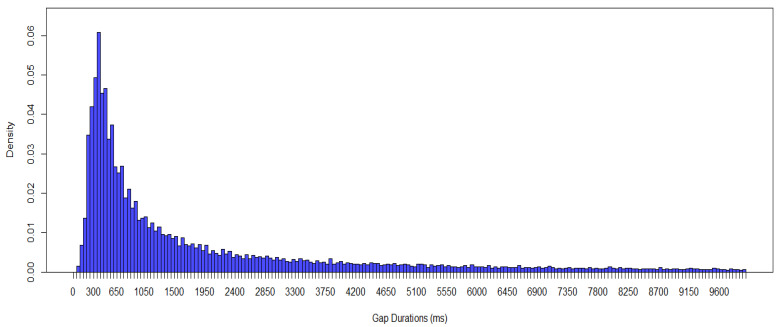
Gap Durations Between Protophones in TD Infants. Each bar shows a 50 ms time interval.

In our investigation of gap durations between protophones in 103 TD infants (1067 five min segments), the data showed predominantly short gap durations, under 1000 ms, with a mode at the 351–400 ms interval (see Figure 1). 500 ms is the duration of a long infant syllable [29], so infants seemed to produce the next protophone in a cluster quickly.

**Figure 2:**
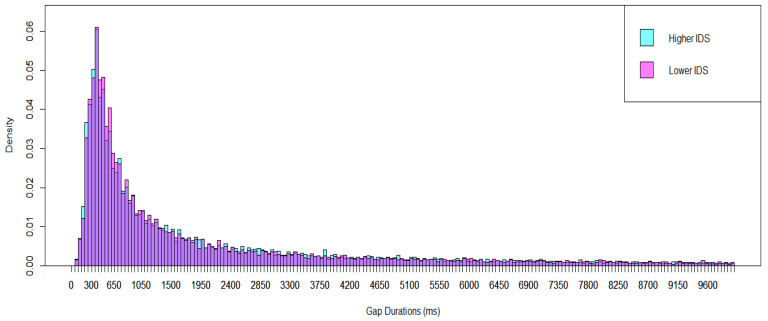
Breakdown of the Results of TD Infants into Two Groups of Recording Segments, Higher and Lower IDS. The purple color represents the overlapping region of the two groups.

We broke down the results into two groups of recording segments based on the amount of infant-directed speech (IDS) occurring in each 5 min segment: (1) lower IDS segments that included approximately 14% of adult utterances directed to infants (532 five min segments), and (2) higher IDS segments that included approximately 45% of adult utterances directed to infants (535 five min segments, See the Supporting Information of [25] for the automated algorithm for higher and lower IDS selection). The results showed similar modes for both IDS conditions (351–400 ms). The Kolmogorov–Smirnov test showed a significant difference in the distributions of gap durations between higher and lower IDS in TD infants (*p* = 0.001; see Figure 2).

#### 3.1.2. Gap Durations Between Protophones in TD and Autistic Infants in Higher and Lower IDS Recording Segments

**Figure 3:**
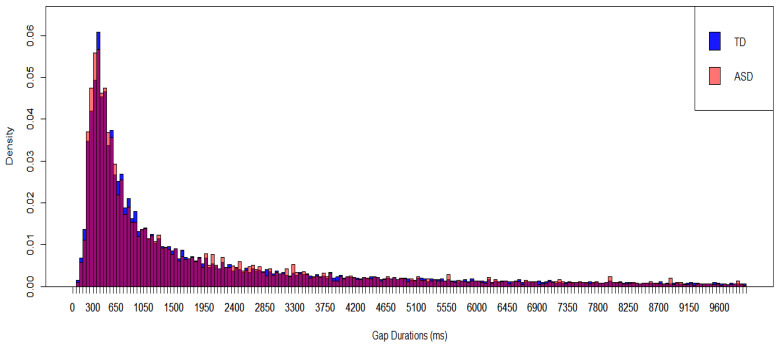
Gap Durations Between Protophones in TD Infants and Autistic Infants. The dark purple color represents the overlapping region of the two groups. Figure 3 presents data collapsing across segments with higher and lower IDS.

The data on 44 autistic infants (431 five min segments) showed that the ASD infants predominantly exhibited short gap durations (similar to the TD infants), with a mode between 351 and 400 ms for data collapsed across higher and lower IDS segments (see Figure 3). The Kolmogorov–Smirnov test showed no significant difference in the distributions of gap durations between the ASD and TD groups (*p* = 0.137) across the whole dataset when the results were not broken down by higher and lower IDS segments.

**Figure 4:**
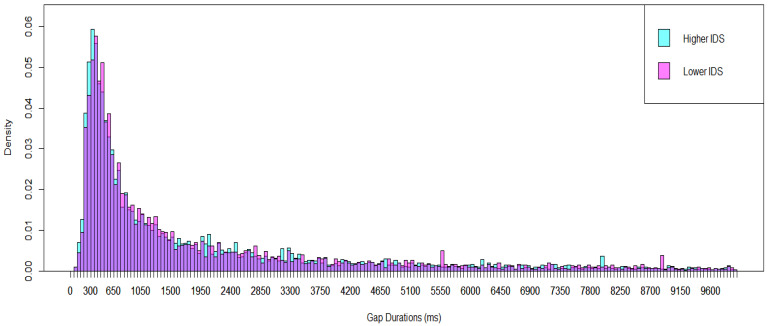
Breakdown of the Results of Autistic Infants into Two Groups of Recording Segments, Higher and Lower IDS. The purple color represents the overlapping region of the two groups.

In the ASD group, the mode for higher IDS segments (including approximately 53% of adult utterances directed to infants) was 301–350 ms, and for lower IDS segments was 351–400 ms (including approximately 18% of adult utterances directed to infants; see Figure 4). The Kolmogorov–Smirnov test showed a significant difference in the distributions of gap durations between higher and lower IDS in autistic infants (*p* = 0.030).

We also compared the ASD and TD groups across lower and higher IDS segments separately. There was a significant difference between ASD and TD groups in the distributions of gap durations for the higher IDS segments according to the Kolmogorov–Smirnov test (*p* = 0.006). However, there was no significant difference in the distribution of gap duration for the lower IDS segments (*p* = 0.509) between groups.

#### 3.1.3. Gap Durations Between Protophones Across Age Groups in TD and ASD Groups

As part of our third study question, we grouped the ASD and TD groups into three different age groups: 0 to 3 months, 4 to 7 months, and 8 to 12 months. Most gap durations between protophones were very short at all three age ranges in both groups. Table 2 shows a summary of the mode for different age groups in ASD and TD groups.

The Kolmogorov–Smirnov test showed significant differences in the distributions of gap durations across age groups in both autistic and TD infants. In the TD group, gap duration distributions differed significantly between 0 and 3 months and 4–7 months (*p* < 0.001), 0–3 months and 8–12 months (*p* < 0.001), and 4–7 months and 8–12 months (*p* < 0.001). In the ASD group, gap duration distributions also showed significant differences between 0 and 3 months and 4–7 months (*p* = 0.039), 0–3 months and 8–12 months (*p* < 0.001), and 4–7 months and 8–12 months (*p* < 0.001). The findings indicate that gap durations changed with age. Although the K-S test did not allow direct comparison across the groups for gap patterns with age, we noted that the patterns appeared different; the mode reduced across age for TD infants, but increased for ASD infants, a pattern that will be revisited in the GLM analysis below.

#### 3.1.4. Gap Durations Between Protophones Across the Sexes in TD and Autistic Infants

**Figure 5:**
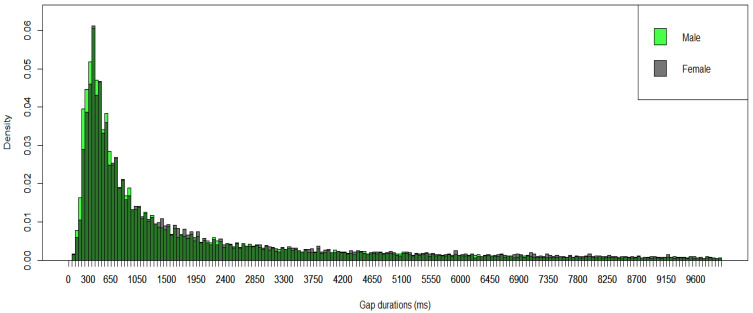
Gap Durations between protophones in TD Infants across sexes. The dark green color represents the overlapping region of the two groups.

**Figure 6:**
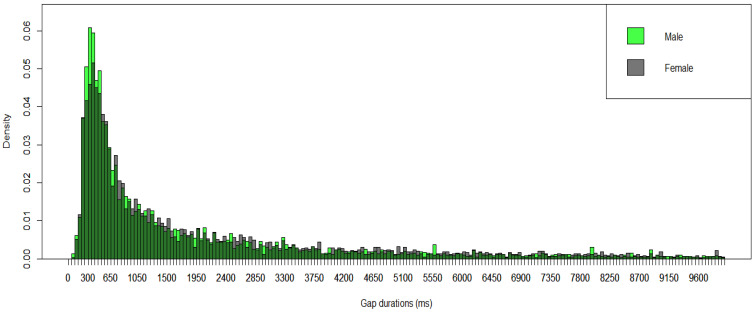
Gap Durations Between Protophones in Autistic Infants across Sexes. The dark green color represents the overlapping region of the two groups.

Oller et al. [30] showed there is a significant difference in volubility (number of protophones per minute) between males and females in the first year of life. In an additional study, it was found that autistic males had lower canonical babbling ratios (CBR, number of canonical syllables as a proportion of total syllables, canonical and noncanonical) than TD males, but females showed similar CBR across groups [31]. To build on these findings, we evaluated gap durations between protophones in both TD and autistic infants across sexes using the K-S test. Again, most gap durations between protophones were very short across both sexes in the ASD and TD groups, with a mode at the 351–400 ms interval for TD infants and for autistic females. However, the autistic males were different, showing a mode at the 301–350 ms interval (see Figure 5 and Figure 6).

We found a significant difference according to the Kolmogorov–Smirnov test (*p* < 0.001 for the TD group, and *p* < 0.001 for the ASD group) in gap durations between protophones across males and females in both groups.

### 3.2. Generalized Linear Modeling of Gap Durations

**Figure 7:**
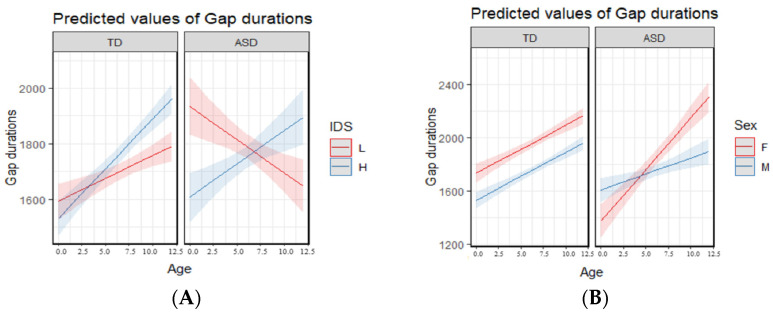
Generalized Linear Model results for three-way interactions. Error bars and shading ranges represent 95% confidence intervals (Note: H = Higher IDS, L = Lower IDS, TD = Typically Developing, ASD = Autism Spectrum Disorder, F = Female, M = Male). (**A**): IDS × Diagnosis × Age. (**B**): Sex × Diagnosis × Age.

**Figure 8:**
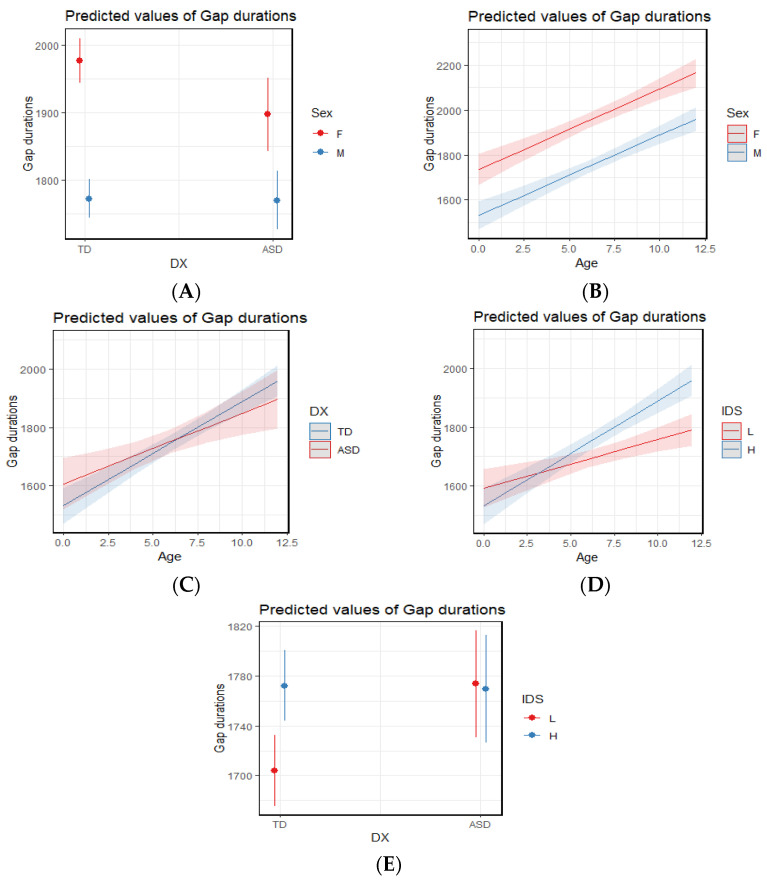
Generalized Linear Model results for two-way interactions. (**A**): Sex × Diagnosis. (**B**): Sex × Age. (**C**): Diagnosis × Age. (**D**): IDS × Age. (**E**): IDS × Diagnosis.

To explore potential main effects and interactions between diagnosis (TD vs. ASD), IDS (higher vs. lower), sex, and age on the duration of gap durations between protophones, we conducted a generalized linear model (GLM) analysis based on the distribution of gap durations, focusing on predicted means and confidence intervals. The model revealed several significant three-way and two-way interactions (see Table 3), including IDS × Diagnosis × Age (Figure 7A), Sex × Diagnosis × Age (Figure 7B), Sex × Diagnosis (Figure 8A), Sex × Age (Figure 8B), Diagnosis × Age (Figure 8C), IDS × Age (Figure 8D), and IDS × Diagnosis (Figure 8E). The results also showed that all four main effects in the GLM analysis were significant (see Table 3). 

The main effects were as follows: The TD infants exhibited longer gap durations than the autistic infants, with a very small effect size (predicted mean: TD = 1838, ASD = 1832; 95% CI for TD = [1820, 1855], for ASD = [1804, 1861]; Cohen’s *d* = 0.06). Segments with higher IDS showed longer gap durations than segments with lower IDS (predicted mean: high IDS = 1849, low IDS = 1821; 95% CI for high IDS = [1826, 1873], for low IDS = [1797, 1844]; Cohen’s *d* = 0.19). Females showed longer gap durations than males, with a very large effect size (predicted mean: females = 1917, males = 1754; 95% CI for females = [1889, 1944], for males = [1734, 1773]; Cohen’s *d* = 1.62). Gap durations were longer for older infants (age 6–12 months) than for younger infants (age 0–5 months), also with a very large effect size (predicted mean: older = 1902, younger = 1754; 95% CI for older = [1879, 1925], for younger = [1731, 1777]; Cohen’s *d* = 1.07).

As previously noted, there were several significant two-way and three-way interactions (see Table 3 and Figure 7 and Figure 8). Rather than presenting each interaction individually, we organize the findings thematically—by sex, age, IDS, and diagnosis to highlight meaningful effects and reveal interesting patterns that were not revealed based on main effects alone.

#### 3.2.1. Interactions Related to Sex Differences

Across both TD and ASD groups, females exhibited longer gap durations than males. This sex difference remained consistent across age ranges (see Figure 8B). Also as shown in Figure 8A, the effect was much larger in the TD group than in the ASD group.

A related three-way interaction (Diagnosis × Sex × Age, Figure 7B) revealed an interesting developmental pattern: autistic females began with shorter gap durations but showed a sharp increase over time, eventually surpassing TD females after 9 months. This pattern was not found in males. TD males began with shorter gap durations compared to autistic males but showed increased gap durations more than autistic males across age.

#### 3.2.2. Interactions Related to Age Effects

In older infants, gap durations increased across both diagnostic groups and sexes (see Figure 7B and Figure 8B,C).

The Age × Diagnosis interaction (Figure 8C) also demonstrated an interesting pattern: Autistic infants showed longer gap durations during the earliest months; however, TD infants exhibited a sharper increase in gap durations after 6 months and ultimately showed longer gap than the ASD group. The related two- and three-way interactions (Age × IDS and Age × IDS × Diagnosis, Figure 7A and Figure 8D) revealed additional developmental patterns. The Age × IDS interaction showed that at the youngest ages, lower IDS segments were associated with longer gap durations; however, at older ages, higher IDS segments were associated with longer gap durations. In addition, the Age × IDS × Diagnosis interaction showed that as autistic infants grew older, gap durations reduced in the lower IDS segments. This pattern was not observed in TD infants nor in higher IDS segments of autistic infants.

#### 3.2.3. Interactions Related to IDS Effects

IDS revealed different patterns by diagnostic group. The Diagnosis × IDS two-way interaction (Figure 8E) showed that TD infants showed longer gap durations in higher IDS segments and were more sensitive across IDS. Autistic infants, however, demonstrated very small differences between higher and lower IDS. The Diagnosis × IDS × Age three-way interaction (Figure 7A) also revealed that TD infants showed increased gap durations across age in both higher and lower IDS, with a steeper rise under higher IDS. In contrast, gap durations in autistic infants decreased over time in lower IDS and increased only slightly in higher IDS.

#### 3.2.4. Interactions Related to Diagnosis Effects

Interesting patterns were revealed for interaction with diagnosis. While autistic infants showed longer gap durations in the first six months, TD infants surpassed them after six months (Figure 8C). The diagnosis × IDS two-way interaction showed that autistic infants had minimal change across IDS; however, TD infants exhibited larger gap durations in higher IDS and were more sensitive across IDS (Figure 8E). In the Diagnosis × IDS × Age interaction (Figure 7A), this pattern was even more noticeable, as TD infants showed increasing gap durations with time across IDS, particularly in higher IDS segments; however, gap durations in lower IDS segments decreased in autistic infants, while they increased more gradually in higher IDS segments.

## 4. Discussion

### 4.1. Summary of Findings

Short gap durations occurred between protophones produced by infants, with a mode between 301 and 400 ms across all groups—typically developing (TD) and autistic infants, both males and females, and the mode was similar at all ages. This suggests that infants predominantly tended to produce protophones in clusters with gaps between them that had just about the durations of individual syllables in infant babbling [29]. However, surprisingly, we found a significant difference with the K-S test in the distribution of gap durations between the ASD and TD groups when infant-directed speech (IDS) was relatively high. This may indicate that TD infants were more responsive to IDS input, whereas infants with ASD were less influenced by such input, potentially reflecting early differences in social-attentional engagement. We also found significant differences with the K-S test in the distribution of gap durations between all age groups and across the sexes.

The GLM findings tended to support the findings from the K-S test but provided additional detail. This model indicated that gap durations were not solely affected by one variable at a time but were shaped by interaction among diagnosis (TD vs. ASD), IDS (higher vs. lower), sex, and age. The overall GLM suggested that longer gap durations were not only associated with TD infants and higher IDS but were also strongly associated with females (Cohen’s *d* = 1.62), and older age (Cohen’s *d* = 1.07). Based on both the raw means (from the data) and the predicted means (from the GLM), the mean gap-duration difference between females and males was approximately 170 ms. The difference between older and younger infants was about 150 ms. These interaction results extend our understanding of how diagnosis, sex, IDS, and age worked together in influencing early vocal timing patterns.

Females had longer gap durations in both the TD and ASD groups. Prior research found that males produced more protophones than females during the first year of life [30]. Higher volubility in males could lead to shorter gaps between protophones due to more frequent vocalizations in a cluster. Social engagement differences could also play a role, suggesting that males are more attracted to the complex stimulation of groups, which leads to producing more continuous vocalizations, while females may engage more in individual interactions in infancy [32].

Longer gap durations at older ages could be associated with a physical change in infant respiration with age [33] or a tendency to be influenced by increasing locomotion [34], either of which might allow longer breath groups, which could influence the structure of protophone clusters. Additionally, the increased mass of articulators with age might also slow vocal movement timing. Another possibility is that protophones become increasingly intentionally communicative with age [7], and this may require mental resources that slow the pace of infant protophone sequences. In addition, as infants grow older, they start to produce a wider variety of sounds and experiment with different pitches and tones [35], which could require more time for inhalation before starting a new sequence of protophones.

### 4.2. Limitations and Future Studies

While the 10 s frame used here does not reveal details in gap durations across hierarchical structures of clustering, this study lays the foundation for more detailed future analyses. Future research could explore varying time frames to identify longer or shorter clusters. Another approach could involve using human listener judgments (as performed by [13]) to validate the time frames and acoustic parameters listeners use in determining the hierarchy of clustering.

We also propose that utterance durations may interact with gap durations (see Supplement S2 about duration of utterances in the present study). Future studies could investigate this relationship by measuring the duration of each utterance and the corresponding gap.

While some statistically significant group differences were found in higher IDS segments, the effect sizes were small. In future research, we could examine interactions where infants and caregivers are both engaged (infants responding to adults) to determine whether infants still produce shorter gap durations in lower IDS than in higher IDS segments. If these findings are replicated with larger effect sizes, the results could provide meaningful insights for clinical approaches. Moreover, analyzing the duration of caregiver utterances and their associated gaps may help determine whether caregiver timing patterns influence vocal behavior in infants.

Some recent studies have also used machine learning techniques to analyze infant vocalizations for the early detection of ASD [36,37]. Including these methods in future studies may provide new insights into the prediction of vocal developmental patterns in autistic infants.

### 4.3. Importance of the Study

This study is the first to ask how far apart protophones are within an infant cluster produced at home throughout the day. Thus, we report on a question not previously addressed in the literature. This approach provides an overall perspective on how infant utterances are organized in time. Lynch et al. [13] used listener judgments to define infant vocal clustering hierarchically at several levels of temporal organization: syllables (the minimal rhythmic units of the system) can be grouped within utterances (which constitute the second level of the rhythmic hierarchy), and these utterances can then be grouped within larger units that can be called “clusters” or prelinguistic phrases (the third level). The human listeners in the study also recognized a fourth level, the supercluster. However, in the present work, we used a fixed 10 s criterion for the whole-time frame to evaluate gap durations between protophones. The decision was arbitrary; a shorter or longer total frame could have been chosen. It is evident that if the maximum duration of the time frame is decreased, the mean is necessarily shortened. Importantly, because we decided on a long interval, we may have encompassed all 4 levels of the hierarchy mentioned in [13]. Consequently, the approach did not differentiate between the levels. But the modal gap duration of 301–400 may reflect typical modal gap durations for any level of the hierarchy. By using a single long frame, we were able to compare various factors (age, sex, ASD vs. TD, and higher or lower IDS) to compare gap duration means. This pattern we found suggests that the temporal organization of infant vocal development may be substantially structured across the different variables. The work lays the foundation for future research aimed at determining cluster organization. In addition, measuring the mean duration between protophones in a cluster is a conservative approach that helps quantify how far apart protophones are spaced on average and suggests surprising interactions among factors, with sex playing a particularly important role.

## 5. Conclusions

This study investigated the temporal organization of infant vocalizations in a naturalistic setting, focusing on the gap durations between protophones. Our findings revealed that infants, regardless of diagnostic group, sex, and age, predominantly produce protophones with short gap durations between them, roughly the length of a long infant syllable. While the modes of gap durations between groups were consistent, we found significant differences in gap duration distributions between ASD and TD groups when infant-directed speech was more frequent, as well as across age groups and sexes. Generalized linear modeling confirmed these findings and revealed the most prominent effects were seen for sex (females showing longer gaps than males) and age (older infants producing longer gaps than younger ones) in both ASD and TD groups. By finding gap durations in clusters of infant vocalizations, we highlighted the potential of temporal structure as an early developmental marker. The results provide new insights into the timing organization of prelinguistic vocal behavior and set the stage for future exploration in this field.

## Figures and Tables

**Table 1:** Summary of studies assessing the mean of intrapersonal pauses in infancy during interaction sessions.

Study	Northrup and Iverson, 2015 [19]	Gratier et al., 2015 [20]	Jaffe et al., 2001 [2]
N	HR: 25 LR: 10	51	88
Age	HR: 5–14 mo. LR: 2–19 mo.	8–21 weeks	4 mo.
Means of Intrapersonal pauses (ms)	HR: 2000 ms LR: 1800 ms	745 ms (Range = 300 to 3000 ms)	MI: 660 ms SI: 520 ms

Note: HR = High risk for ASD, LR = Low risk for ASD, MI = Mother–infant interaction, SI = Stranger–infant interaction.

**Table 2:** Summary of mode for different age groups in ASD and TD groups (in milliseconds).

Age Range	TD Mode	ASD Mode
0–3 mo.	351–400	301–350
4–7 mo.	351–400	351–400
8–12 mo.	301–350	351–400

**Table 3:** GLM regression coefficients predicting gap duration.

Predictor	B	SE	t-Statistic	*p*
(Intercept)	1377.582	64.160	21.471	<0.001
DX (TD vs. ASD)	358.400	73.127	4.901	<0.001
IDS (Low vs. High)	328.433	63.614	5.163	<0.001
Sex (Male vs. Female)	229.623	68.918	3.332	<0.001
Age	77.030	9.290	8.292	<0.001
DX × IDS	–268.320	74.661	–3.594	<0.001
DX × Sex	–433.703	79.363	–5.465	<0.001
DX × Age	–41.306	10.481	–3.941	<0.001
IDS × Age	–48.140	9.394	–5.124	<0.001
Sex × Age	–52.934	9.925	–5.333	<0.001
DX × IDS × Age	29.058	10.765	2.699	0.007
DX × Sex × Age	52.883	11.252	4.700	<0.001

Note: B = unstandardized coefficient; SE = standard error; *p* values two-tailed; DX = diagnosis; IDS = infant-directed speech. Significance levels: *p* < 0.05; *p* < 0.01; *p* < 0.001.

## Data Availability

The raw audio files contain personally identifiable information and cannot be publicly released under HIPAA privacy regulations. However, upon reasonable request, the corresponding author can provide de-identified datasets supporting this study’s results. The recordings were all made at the Marcus Autism Center in Atlanta. Their confidentiality provisions are specified in the Emory University IRB approval. The recordings to be coded in Memphis were transmitted to the OLL administrators (Oller and Bene) securely, with no identifying information for any subject provided. Thereafter, 21 five min segments were selected at random from each recording by an OLL automated procedure. These were the only segments that were coded for the reported data. All coders, including research staff, were blinded with regard to linkage of recordings, demographic, and diagnostic data. No family or individual identification was provided to any member of the OLL team. The recordings themselves were stored on a designated data drive in Memphis, which was encrypted and password protected. The data drive with the selected segments was only accessible for coding to OLL coders. Only research staff had access to full recordings, and only for the period of time until coding was completed. After coding was complete, full recordings were deleted from OLL drives and became inaccessible for further segment selection in Memphis. Each segment/recording/infant was marked with a specific UofM study ID.

## Supplementary Materials

The following supporting information can be downloaded at: https://www.mdpi.com/article/10.3390/brainsci15080819/s1, Supplement S1: Visual Representation of the Gap Durations Between Protophones and Clustering Pattern; Figure S1: Gap Durations between Protophones; Figure S2: Gap Durations between Protophones with Intervening Cry or Whimper; Figure S3: Gap Durations between Protophones with Intervening Utterances of Other Speakers; Supplement S2: Duration of utterances; Table S1: Summary of the mean and standard deviation of utterance duration across different conditions (in milliseconds).
