# Supplementary material for: Timing of Intervals Between Utterances in Typically Developing Infants and Infants Later Diagnosed with Autism Spectrum Disorder
Source: Brain Sci. 2025 Jul 30;15(8):819. doi: 10.3390/brainsci15080819 (PMC12384503; doi:10.3390/brainsci15080819)
Supplement: Supplementary file 1 [file brainsci-15-00819-s001.zip › brainsci-3735432-supplementary.pdf]

Supplement S1: Visual Representation of the Gap Durations Between Protophones in Clusters:

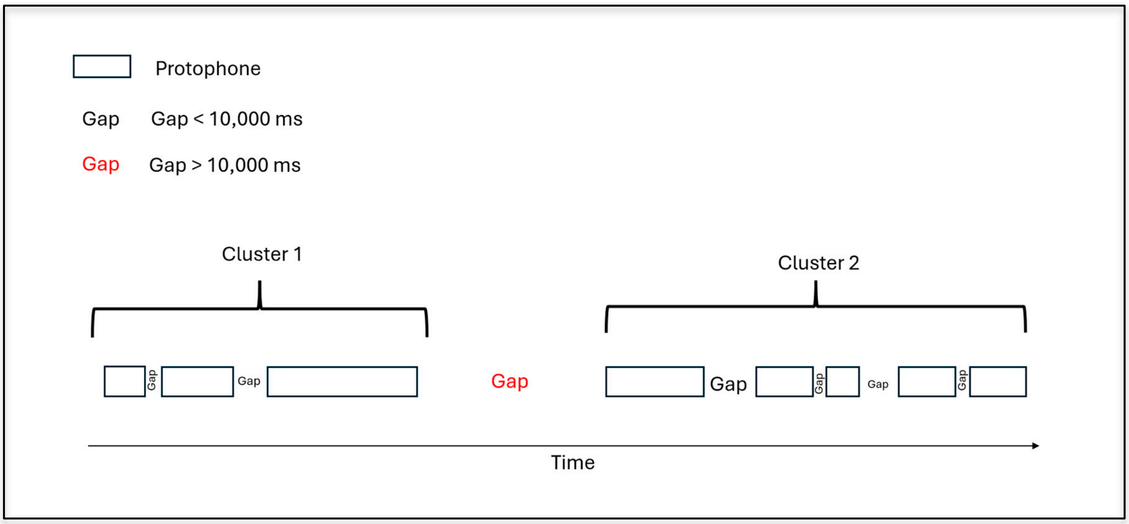

Figure S1. Gap Durations between Protophones.

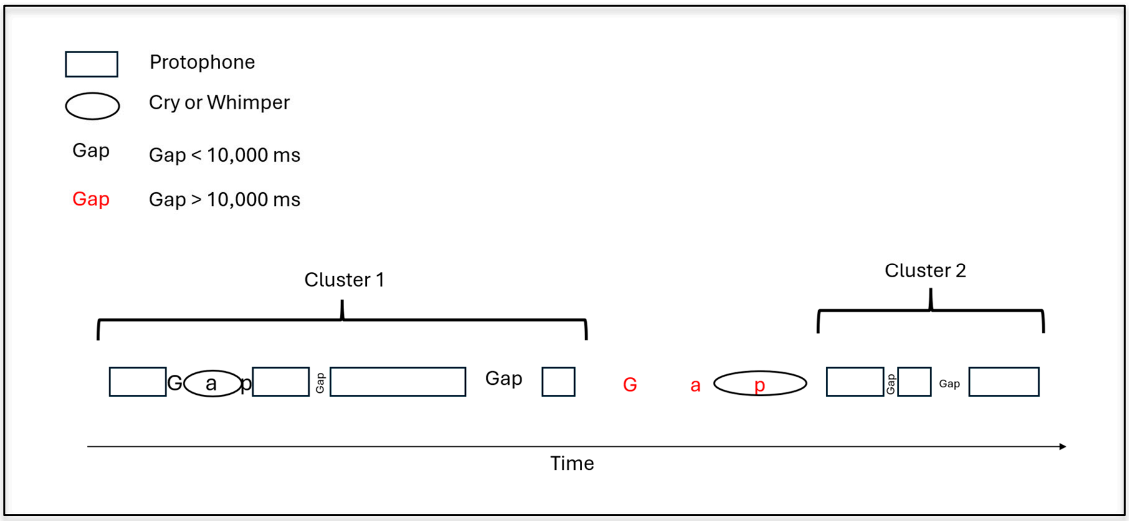

Figure S2. Gap Durations between Protophones with Intervening Cry or Whimper. Cries and whimpers were simply ignored in the measurements determining gaps between protophones.

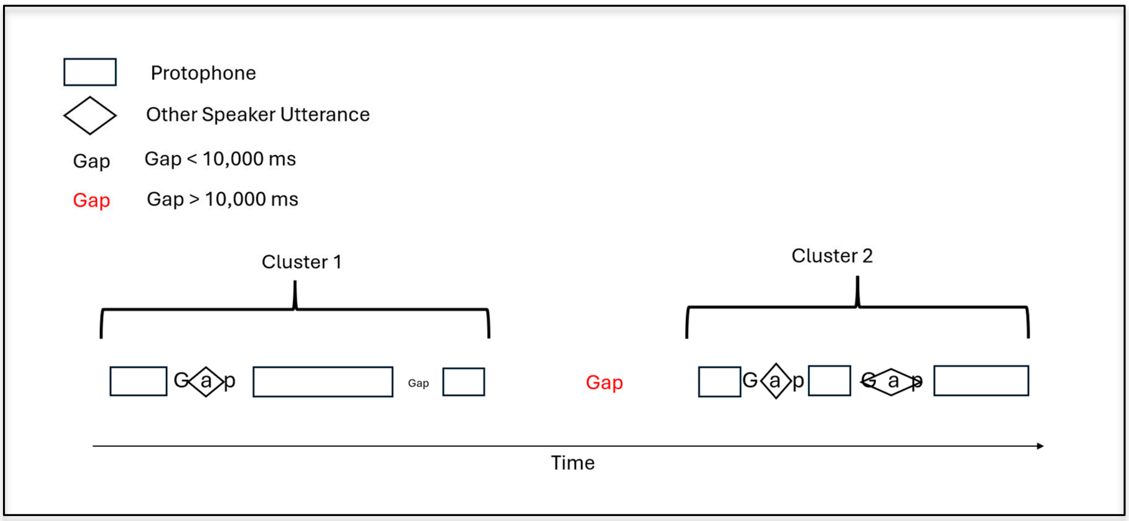

Figure S3. Gap Durations between Protophones with Intervening Utterances of Other Speakers. Intervening utterances of other speakers were simply ignored. Thus, the end of one protophone to the beginning of the next protophone was measured as a gap duration regardless of whether an intervening utterance of another speaker or a cry or laugh by the infant occurred between them.

Note: All short gap durations < 75 ms and all gap duration > 10,000 ms were removed from the data at the point of analysis (see Data Analysis in the Method section for more details).

Supplement S2: Duration of utterances

We measured durations of utterances across groups (see table A1), but we do not know what the relation was between duration of each utterance and the following gap. Future studies in our laboratory will investigate this relationship by measuring the duration of each utterance and the corresponding gap.

Table S1. Summary of the mean and standard deviation of utterance duration across different conditions (in milliseconds).

	0-3 mo.	4-7 mo.	7-12mo.	Boys	Girls	Higher IDS	Lower IDS
TD	650 (216)	881 (288)	922 (246)	813 (170)	887 (239)	827 (225)	861 (223)
ASD	681 (162)	875 (237)	913 (219)	846 (197)	862 (178)	877 (254)	823 (199)
